# Exploring the Anti-Cervical Cancer Effect and Hepatotoxicity Risk of Gossypol Based on Untargeted Metabolomics and Network Toxicology

**DOI:** 10.3390/ph19030377

**Published:** 2026-02-27

**Authors:** Jinyan Li, Parwen Parhat, Yinglan Ma, Liuqian Peng, Min Li

**Affiliations:** 1 State Key Laboratory of Pathogenesis, Prevention and Treatment of High Incidence Diseases in Central Asia, College of Pharmacy, Xinjiang Medical University, Urumqi 830011, China; jinyanli1015@163.com (J.L.); 18371980618@163.com (P.P.); myl2443382113@163.com (Y.M.); 13150350230@163.com (L.P.); 2 Xinjiang Key Laboratory of Natural Medicines Active Components and Drug Release Technology, Urumqi 830011, China; 3 State Key Laboratory of Natural Medicines, China Pharmaceutical University, Nanjing 210009, China

**Keywords:** gossypol, cervical cancer, untargeted metabolomics, network toxicology

## Abstract

**Objectives**: This research sought to examine the impact of gossypol on cervical cancer tumors that have been transplanted subcutaneously in nude mice, as well as the associated risk of liver damage and its underlying mechanisms. **Methods**: A subcutaneous cervical cancer tumor model was established in nude mice using the cell suspension inoculation method. Tumor volume and morphological changes in various organs were observed, and the serum concentrations of IL-6, IL-10, and TNF-α were assessed. Protein expression was analyzed using Western blotting. Untargeted metabolomics was employed to identify differential metabolites in mouse liver tissues. Network toxicology was utilized to pinpoint common targets associated with gossypol and hepatotoxicity, followed by KEGG and GO enrichment analyses. Molecular docking was conducted to preliminarily explore the mechanisms underlying gossypol-induced liver injury. **Results**: Gossypol significantly suppressed the development of subcutaneous cervical cancer tumors in immunodeficient mice. The Western blotting technique results revealed that increasing doses of gossypol led to a reduction in the expression levels of PIK3R2, GRB2, and MAPK1, compared to the model group (*p* < 0.05). Untargeted metabolomics revealed 1464 metabolites, from which 9 distinct metabolites were selected for further analysis. Network toxicology results indicated that the hepatotoxicity-related targets of gossypol included MTOR, TNF, CASP3, BCL2L1, and BCL2. KEGG analysis suggested that the toxic mechanisms may be linked to pathways involved in malignancy, the HIF-1 signaling pathway, proteoglycans in cancer, apoptosis, and others. **Conclusions**: Gossypol demonstrates a significant therapeutic effect against cervical cancer; however, its hepatotoxicity risk, mediated through multiple targets and pathways, requires further investigation.

## 1. Introduction

Cervical cancer (CC) is the fourth-most common malignant tumor affecting women globally, with around 660,000 new cases and 350,000 fatalities documented in 2022. The incidence and mortality rates are particularly pronounced in low- and middle-income countries [1]. This disease is mainly caused by a persistent infection with human papillomavirus (HPV) and is considered a preventable and manageable malignancy due to the availability of effective vaccines and screening methods [2]. Nevertheless, cervical cancer remains one of the primary causes of cancer-related deaths in women residing in less developed regions. Although vaccination and screening efforts have diminished incidence rates in certain countries, the rate among women aged 30–44 in the United States increased by 1.7% annually from 2012 to 2019 [3,4]. For early-stage and locally advanced cases, the standard approach to treatment includes a comprehensive hysterectomy accompanied by the dissection of pelvic lymph nodes, often accompanied by adjuvant radiotherapy and/or chemotherapy. Alternatively, the treatment of late-stage cervical cancer poses significantly greater challenges, as systemic therapy and radiotherapy provide only limited survival benefits, despite extending overall survival [5]. Consequently, a more profound investigation regarding the biological processes underlying cervical cancer and the advancement of safer, more effective therapeutic agents are critical and urgent research priorities.

**Figure 1:**
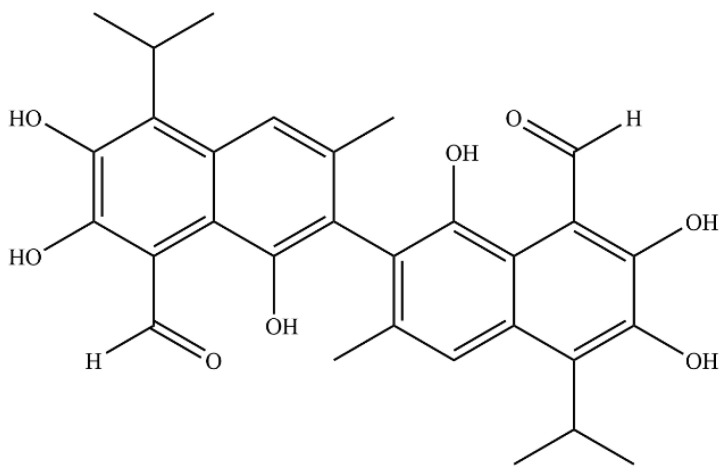
The structure of gossypol.

Gossypol (Figure 1) is an inherently existing polyphenol that can be extracted from the seeds, roots, and stems of land-based cotton plants. It is especially concentrated in cottonseed oil and its by-products, particularly cottonseed meal [6]. This compound with the molecular formula C_30_H_30_O_8_ is chemically designated as 2,2-bis(8-formyl-1,6,7-trihydroxy-5-isopropyl-3-methylnaphthalene). It has garnered considerable attention due to its broad spectrum of pharmacological effects, which include antiviral [7], antioxidant [8], antiparasitic [8], antimalarial [9], and antitumor effects [10,11]. In clinical practice, gossypol is employed in the treatment of gynecological disorders, including uterine fibroids, endometriosis, and abnormal uterine bleeding [12]. Additionally, its derivatives demonstrate potential therapeutic efficacy against various malignancies, including leukemia, lymphoma, colon cancer, breast cancer, prostate cancer, and non-small cell lung cancer, thereby establishing gossypol as a promising candidate to promote the development of novel cancer-fighting substances [13,14]. Our previous studies at the cellular level have confirmed that gossypol exerts anti-cervical cancer effects by modulating the PI3K/AKT signaling pathway, a mechanism closely associated with the expression levels of three key proteins: PIK3R2, GRB2, and MAPK1. While current research suggests that gossypol may be effective in treating cervical cancer [14], research in this field is still quite limited, and the exact mechanisms by which it exerts its effects remain largely unclear. Consequently, the current analysis adopts an integrated approach that combines untargeted metabolomics and network toxicology to systematically elucidate the anti-cervical cancer effects of gossypol while concurrently evaluating its hepatotoxicity risk. This strategy seeks to establish a conceptual framework for utilizing gossypol in therapeutic applications for cervical cancer.

## 2. Results

### 2.1. Effect of Gossypol on the Tumor Growth Inhibition Rate in Nude Mouse Xenograft Models

**Figure 2:**
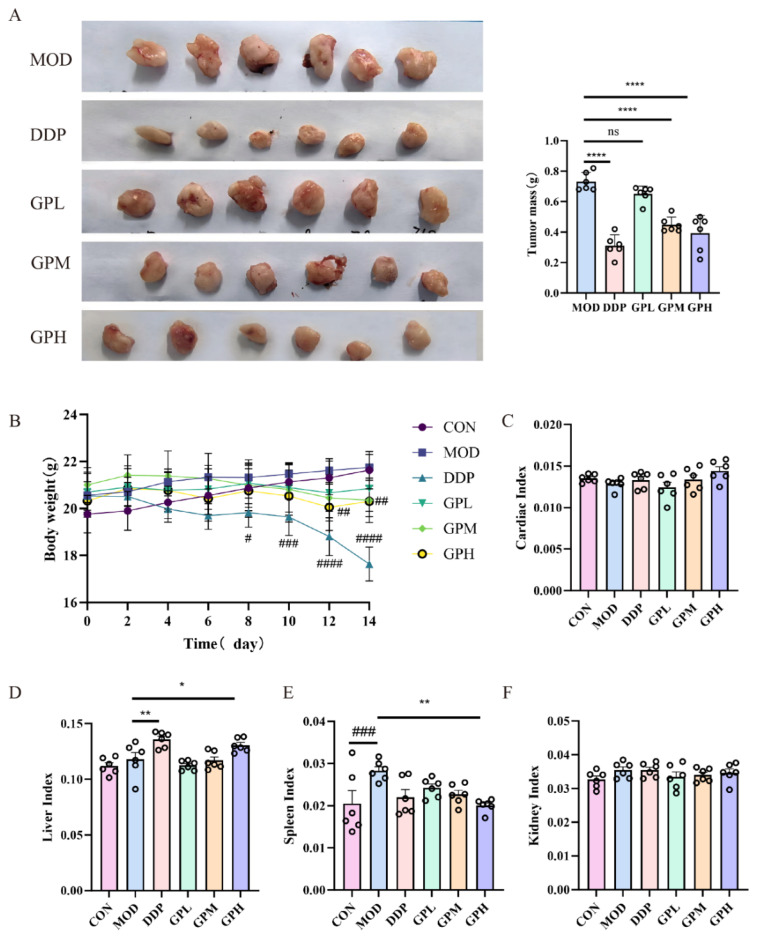
(**A**) Tumor size and weight within each group of mice. (**B**) Changes in body weight. (**C**) Cardiac index. (**D**) Liver index. (**E**) Spleen index. (**F**) Kidney index. ns denotes that there is no statistically significant difference. # *p* < 0.05, ## *p* < 0.01, ### *p* <0.001, #### *p* <0.0001 vs. CON, *n* = 6. * *p* < 0.05, ** *p* < 0.01, **** *p* <0.0001, vs. MOD, *n* = 6.

After 14 days of treatment, compared with the model group, the cisplatin group (DDP) and the medium- and high-dose gossypol groups (GPM, GPH) exhibited significantly reduced tumor mass (*p* < 0.0001). The corresponding tumor inhibition rates were 57.63%, 38.72%, and 46.24%, respectively (Table 1, Figure 2A).

### 2.2. Effect of Gossypol on Mouse Body Weight and Organ Indices

In comparison to the control group, mice in both the cisplatin and high-dose gossypol groups exhibited a reduction in body weight, with the decrease being more pronounced in the cisplatin group (Figure 2B). Assessment of organ indices revealed no significant alterations in the cardiac index across all groups (Figure 2C). Both the cisplatin and high-dose gossypol groups showed a significant increase in the hepatic index compared to the model group (*p* < 0.05) (Figure 2D). The splenic index was significantly elevated in the model group relative to the control group (*p* < 0.001), whereas it was significantly reduced in the high-dose gossypol group compared to the model group (*p* < 0.01) (Figure 2E). No significant differences were observed in the renal index among the groups (Figure 2F).

### 2.3. Histopathological Analysis of Mouse Tissues

**Figure 3:**
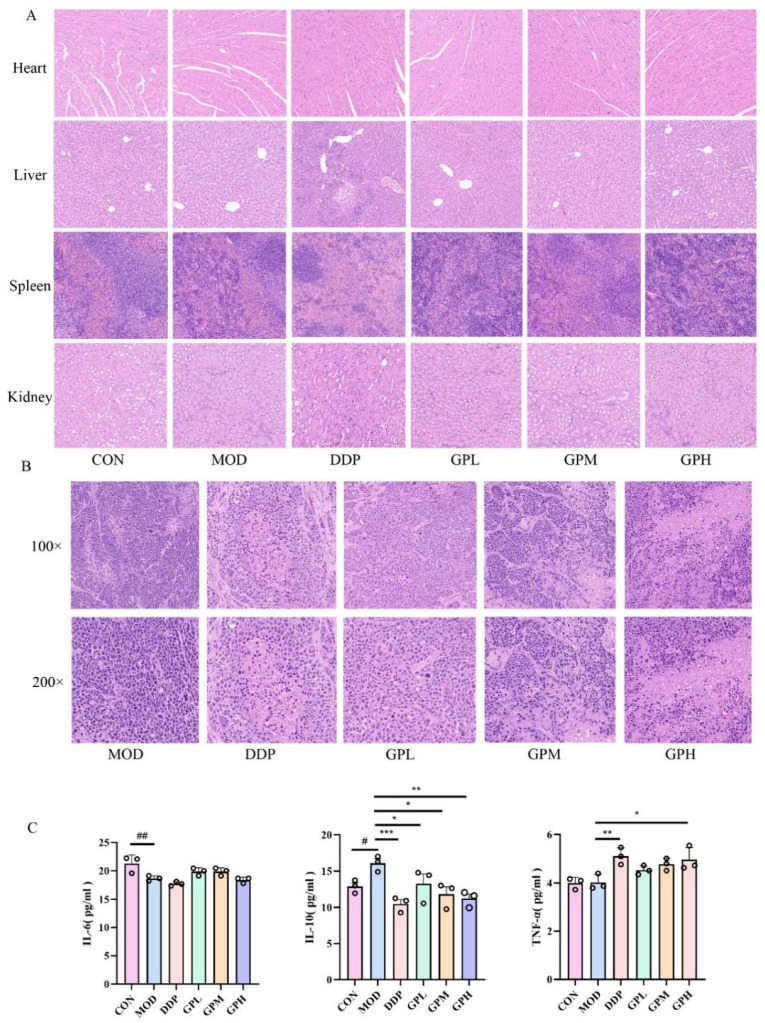
(**A**) Histomorphological changes in various tissues of mice from each group (100× magnification). (**B**) Morphological alterations in tumor tissues across different groups. (**C**) Serum levels of IL-6, IL-10, and TNF-α in mice from each experimental group. ns indicates no significant difference. # *p* < 0.05, ## *p* < 0.01, vs. CON, *n* = 3. * *p* < 0.05, ** *p* < 0.01, *** *p* <0.001, vs. MOD, *n* = 3.

Pathological alterations in major mouse organs were examined via HE staining (Figure 3A). These results indicated normal tissue-related architecture in the heart and kidney tissues across all experimental groups, suggesting no evident cardiotoxicity or nephrotoxicity associated with cisplatin or gossypol administration. In liver tissues, the control group exhibited normal morphology. In contrast, the cisplatin group showed marked hepatocellular necrosis and hemorrhage, while the high-dose gossypol group displayed only minor hemorrhage without necrosis. Splenic pathology revealed intact architecture with clear boundaries and dense lymphocyte populations in both the control group and all gossypol-treated groups. The cisplatin group, however, presented with local hemorrhage and amyloid deposition. Tumor histopathology assessment (Figure 3B) demonstrated that the model group had high tumor cell density with regular morphology. Conversely, the DDP group and GPM and GPH groups exhibited reduced tumor cell density, uneven distribution, and noticeable morphological heterogeneity.

### 2.4. Effects of Gossypol on Serum IL-6, IL-10, and TNF-α Levels in Mice

When compared to the control group, the model group showed a considerable reduction in IL-6 levels (*p* < 0.01), accompanied by a notable increase in IL-10 levels (*p* < 0.05). As opposed to the model group, the IL-10 levels were markedly reduced in the DDP group as well as in the GPL, GPM, GPH groups (*p* < 0.05). Furthermore, there was a notable elevation in TNF-α levels observed in both the DDP group and the GPH group compared to those in the model group (*p* < 0.05) (Figure 3C).

### 2.5. Impact of Gossypol on the Expression Levels of Related Proteins

**Figure 4:**
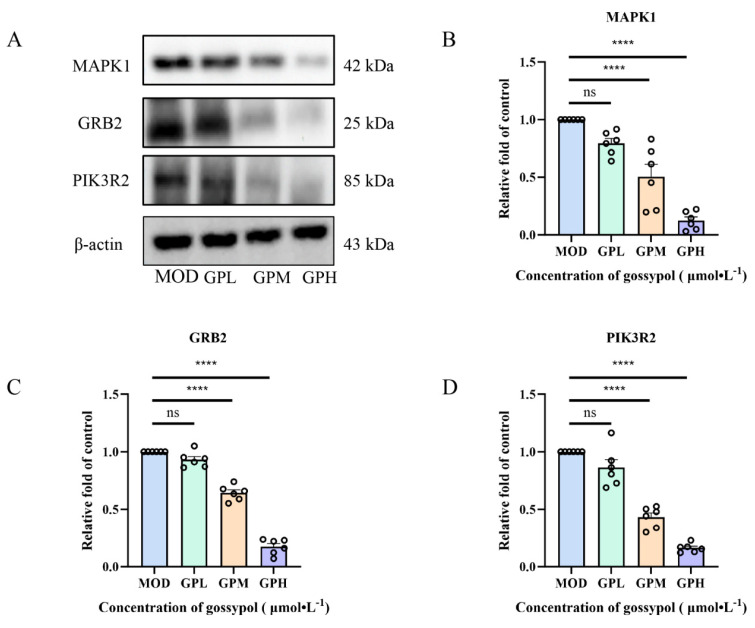
(**A**) Representative immunoblot images of protein bands. (**B**–**D**) Influence of gossypol on the expression of MAPK1, GRB2, PIK3R2 protein. ns denotes that there is no statistically significant difference. **** *p* <0.0001, vs. MOD, *n* = 6.

Following gossypol administration, the protein expression levels of MAPK1, PIK3R2, and GRB2 decreased in a manner dependent on the dosage (Figure 4A). In contrast with the model group, the medium- and high-dose gossypol treatments significantly downregulated the relative expression levels of all three proteins, with the differences being statistically significant (*p* < 0.0001) (Figure 4B–D).

### 2.6. Untargeted Metabolomics Analysis

**Figure 5:**
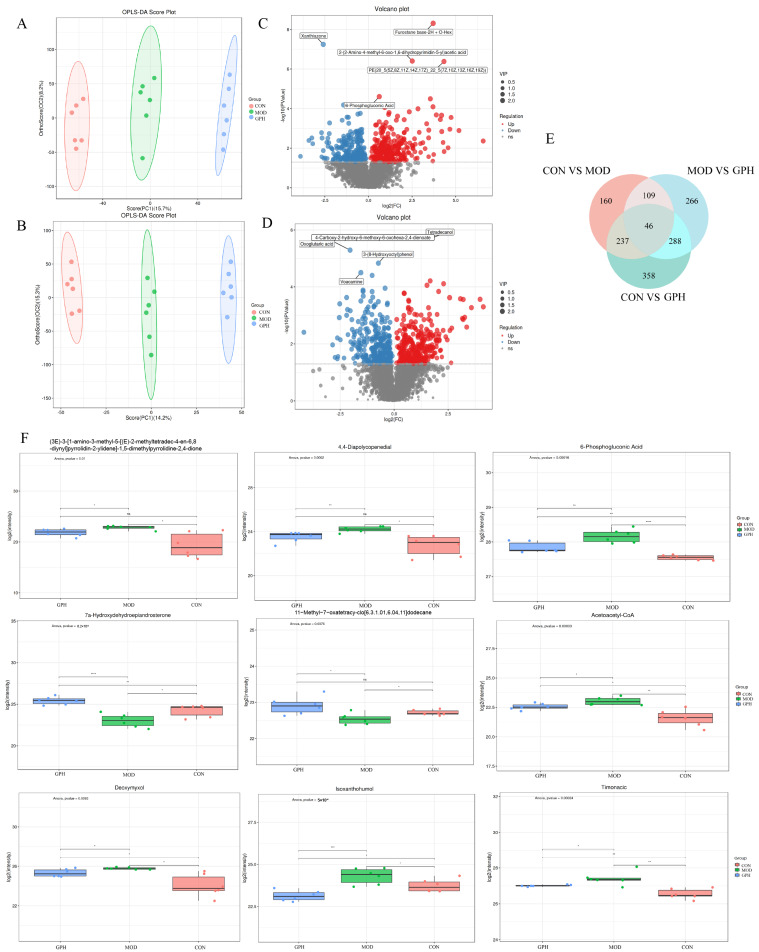
(**A**) Score plot of OPLS-DA (+), (**B**) Score plot of OPLS-DA (−), (**C**) Volcano plot of CON vs. MOD, (**D**) Volcano plot of MOD vs. GPH, (**E**) Venn diagram, (**F**) Level changes in differential metabolites. ns indicates no significant difference. * *p* < 0.05, ** *p* < 0.01, *** *p* < 0.001, **** *p* < 0.0001, *n* = 6.

Data acquisition and processing were performed on samples from the control, model, and treatment groups using LC-MS/MS analysis to investigate changes in hepatic metabolites following model establishment and drug administration. The OPLS-DA results (Figure 5A,B) demonstrated clear separation of the confidence ellipses across the three populations assessed in both positive and negative ion settings, suggesting notable in metabolite profiles and substantial metabolic alterations in the mice.

Metabolites with significant differences were identified in accordance with their VIP values from the OPLS-DA model and *p*-values, adhering to the criteria of VIP > 1 and *p* < 0.05. The findings were then illustrated using volcano plots (Figure 5C,D). A cumulative count of 552 metabolites exhibiting significant differences were identified between the control and experimental groups, comprising 311 significantly up-regulated and 241 significantly down-regulated metabolites. Between the model and treatment groups, 709 differential metabolites were found, including 365 significantly overexpressed and 344 significantly underexpressed metabolites. Venn diagram analysis was employed to pinpoint metabolites that were common or unique across the groups (Figure 5E). Through a comparative analysis of the control, model, and therapeutic groups, shared metabolites were identified, and the trends of changes between groups were confirmed, ultimately yielding 9 potential significantly differential metabolites (Table 2, Figure 5F). These differential metabolites were categorized into five classes: organic heterocyclic compounds, lipids and lipid-like molecules, organic oxygen compounds, phenylpropanoids and polyketides, and organic acids and derivatives.

### 2.7. Network Pharmacological Analysis

**Figure 6:**
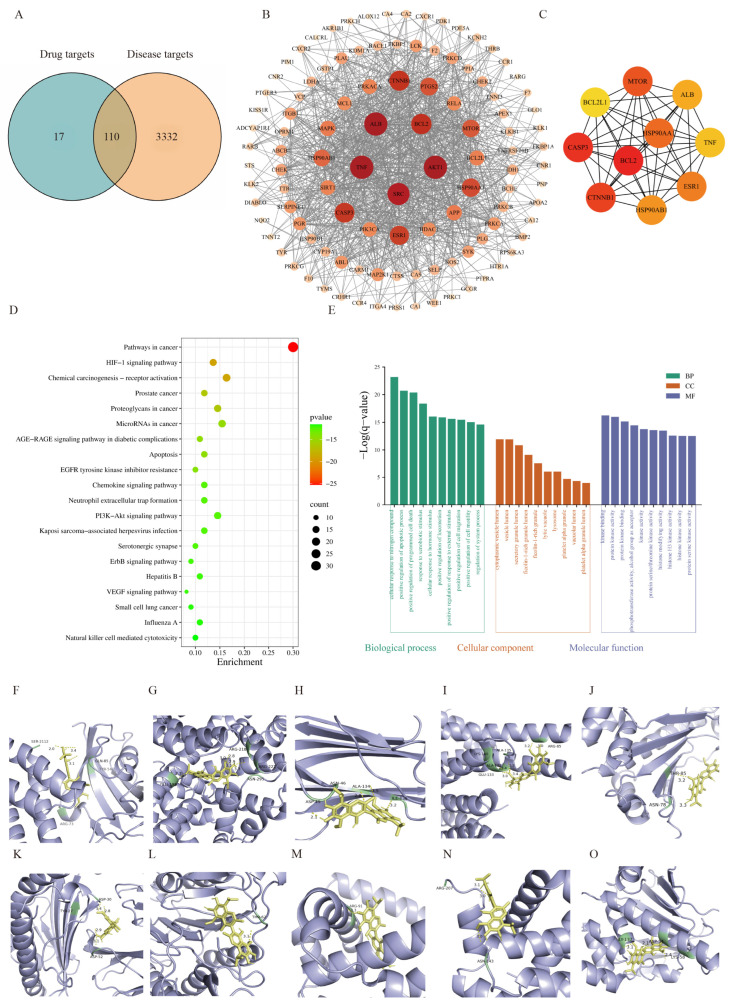
(**A**) Venn diagram of overlapping protein targets between gossypol and hepatotoxicity. (**B**) Protein–protein interaction (PPI) network. (In which, the size and color of a node are determined by the magnitude of its degree: the larger the degree value, the bigger the node and the redder its color.) (**C**) Core targets identified using the Maximal Clique Centrality (MCC) algorithm. (**D**) KEGG analysis. (**E**) GO enrichment analysis. (**F**–**O**) Molecular docking poses illustrating the binding modes of gossypol with key target proteins: mTOR, ALB, TNF, ESR1, HSP90AB1, CTNNB1, CASP3, BCL2L1, BCL2, and HSP90AA1.

The intersection between the identified targets of gossypol and the known targets associated with liver injury was visualized using a Venn diagram, yielding 110 common targets (Figure 6A). A network of protein–protein interactions (PPIs) was developed (Figure 6B), which comprised 109 nodes and 956 edges. The network exhibited an average node degree of 5.83 and an average local clustering coefficient of 0.578. The top 10 targets ranked by Maximal Clique Centrality (MCC) score were mTOR, ALB, TNF, ESR1, HSP90AB1, CTNNB1, CASP3, BCL2L1, BCL2, and HSP90AA1 (Figure 6C), which are suggested to be potential key targets mediating the hepatotoxicity of gossypol. To clarify the physiological roles of these proteins, we performed functional annotation using GO and conducted enrichment analyses of pathways from the KEGG on the 110 targets utilizing Metascape (Figure 6D,E). The KEGG pathway enrichment analysis demonstrated notable links to pathways including pathways in cancer, HIF-1 signaling pathway, proteoglycans in cancer, and apoptosis, all of which are closely related to liver injury mechanisms.

Molecular docking was subsequently carried out to model the interactions between gossypol and the key targets mentioned earlier. The findings indicated that the binding energies for all docked complexes were found to be below −5 kJ·mol^−1^ (Table 3, Figure 6F–O), suggesting a high binding affinity and stable interaction between gossypol and these targets.

## 3. Discussion

This study investigated the inhibitory effect of gossypol on cervical cancer tumors in mice and its potential hepatotoxicity mechanism by integrating untargeted metabolomics and network toxicology. A subcutaneous xenograft mouse model of cervical cancer was first established via inoculation of cell suspension. Observations of tumor volume and morphological changes in various organs revealed that both gossypol and cisplatin exhibited certain inhibitory effects on cervical cancer. Notably, cisplatin caused severe adverse effects on body weight and organs, whereas gossypol demonstrated relatively lower toxicity, consistent with findings from previous studies by Yih-Shou Hsieh [15], Yuling Li [16], Chenglei Ma [17], and Miaomiao Ye [18]. Furthermore, gossypol treatment reduced serum IL-10 levels and elevated TNF-α levels. However, this study has certain limitations. It is important to note that cytokines such as IL-8, IL-1β, and IL-21 also play significant roles in the cervical cancer microenvironment but were not examined here. Consequently, future investigations should employ more clinically relevant models to analyze a broader panel of cytokines, including IL-8 and IL-1β, to comprehensively evaluate gossypol’s remodeling effect on the tumor immune microenvironment. Furthermore, as the tissue origin of these circulating factors cannot be precisely delineated from our current data, subsequent studies should directly measure factor changes within the local tumor microenvironment to establish a more direct causal relationship.

Our previous research indicated that the anti-cervical cancer effect of gossypol primarily involves three proteins of interest, which are PIK3R2, GRB2, and MAPK1 [19]. PIK3R2 is a pivotal element in the PI3K/AKT signaling pathway, essential for promoting cell survival and apoptosis inhibition [20,21]. Evidence suggests that PIK3R2 is overexpressed in various tumors and is closely associated with poor prognosis in certain malignancies [22]. Vera de Geus et al. [23] found that upregulated PIK3R2 expression in cervical cancer ultimately contributes to distant recurrence. GRB2, acting as a critical adaptor protein, connects extracellular signals to intracellular responses, playing a significant role in regulating cell growth, differentiation, and migration [24,25]. Elevated GRB2 levels are observed in multiple cancers, promoting tumor expansion and dissemination [26]. Chin-Chung Lin et al. [27] demonstrated that reducing GRB2 protein levels inhibits the migration and invasion capabilities of cervical cancer HeLa cells. MAPK1, a member of the MAPK family and a crucial element of the MAPK cascade, mediates extracellular signals to regulate diverse cellular processes including proliferation and differentiation. Its activation leads to phosphorylation of transcription factors, thereby promoting inflammatory responses and apoptosis [28,29,30], and it plays an important role in various cancers, including cervical cancer [31]. Xiaowen Li et al. [32] found that interfering with MAPK1 expression significantly suppresses the invasion and metastasis of cervical cancer HeLa cells. This research additionally validated through Western blot analysis that gossypol is capable of suppressing the expression levels of the proteins PIK3R2, GRB2, and MAPK1, indicating that its anti-cervical cancer action is achieved through the regulation of multiple targets.

Untargeted metabolomics was employed to identify major differential metabolites across the control, model, and pharmacological treatment groups. In this study, when assessed against the control group, the model group exhibited a notable increase in the expression levels of 6-phosphogluconate and acetoacetyl-CoA. Following drug intervention, the levels observed in the Administration group demonstrated a significant reversal when compared to those in the Model group. 6-Phosphogluconate is an intermediate product of the pentose phosphate pathway, where it is generated by the oxidation of glucose-6-phosphate catalyzed by glucose-6-phosphate dehydrogenase (G6PD) [33,34]. Relevant studies indicate that the levels of G6PDis increased in tumor cells relative to normal cells, and its expression and activity correlate with the malignancy of diverse types of tumors, including hepatocellular carcinoma [35], malignant peripheral nerve sheath tumors [36], cervical cancer [37], and breast cancer [38]. This suggests that increased 6-phosphogluconate may exacerbate cancer progression. Acetoacetyl-CoA is primarily produced in mitochondria catalyzed by acetyl-CoA thiolase and mainly enters the mevalonate pathway for cholesterol synthesis. Elevated cholesterol transport is a typical characteristic that allows cancer cells to satisfy their membrane biosynthesis needs. In the cytoplasm, it can also be generated directly from acetoacetate via acetoacetyl-CoA synthetase (AACS) [39,40,41]. Studies show that AACS is a highly regulated enzyme expressed in various lipogenic tissues, including tumor tissue [42]. This indicates that increased acetoacetyl-CoA similarly contributes to aggravated cancer progression.

Gossypol intervention led to alterations in the liver index, histopathological damage, and metabolites in mice, suggesting potential hepatotoxic effects. Network toxicology was used to predict its potential molecular targets. Among these, targets such as MTOR, TNF, CASP3, BCL2L1, and BCL2 are highly associated with liver injury. Furthermore, KEGG pathway analysis revealed that the molecular mechanisms of gossypol-induced liver injury might be related to pathways such as pathways in cancer, HIF-1 signaling pathway, proteoglycans in cancer, and apoptosis. In conditions like ischemia–reperfusion liver injury, non-alcoholic steatohepatitis, and alcoholic liver disease, inflammatory factors such as TNF-α, released by Kupffer cells, can directly induce hepatocyte apoptosis or necrosis, leading to liver damage [43,44]. CASP3 is regarded as one of the foremost extensively researched apoptotic protein within the caspase family and is essential for the process of programmed cell death. Its high activation is closely linked to diseases such as myocardial infarction, alcoholic hepatitis, and hepatitis B [45,46]. The proteins encoded by BCL2 and BCL2L1 (Bcl-xL) are important anti-apoptotic molecules that suppress the release of cytochrome c, effectively obstructing the mitochondrial apoptosis pathway [47,48]. MTOR functions as a serine/threonine kinase that regulates cellular metabolism, growth, and autophagy. Furthermore, the MTOR signaling pathway is crucial in the progression of liver damage [49,50]. The hypoxia-responsive core transcription factor HIF-1 and its signaling pathway are key players in processes like hepatic ischemia–reperfusion (IR) injury and fibrosis linked to NAFLD [51,52,53]. Local hypoxia resulting from liver injury can activate this pathway, thereby driving disease progression [54]. Liver injury is characterized by inflammation-mediated hepatocyte apoptosis and necrosis, alongside the release of cytokines that promote inflammation and fibrosis [55,56]. Among these, genetically precisely regulated apoptosis is a core manifestation of this pathological process [57]. Proteoglycans are highly glycosylated proteins widely distributed in the extracellular matrix, present in various tissues, and involved in maintaining tissue homeostasis and microenvironment remodeling [58]. In the liver, they play roles in promoting regeneration and protecting hepatocytes [59,60]. Therefore, while gossypol is effective in treating cervical cancer, it also carries a certain risk of hepatotoxicity, warranting further investigation through molecular biology methods.

## 4. Materials and Methods

### 4.1. Reagents and Chemicals

The study utilized the following chemicals and reagents: gossypol (purity of 99.0% or higher, sourced from Yirui Biotechnology Co., Ltd., Chengdu, China); Matrigel (Corning, Inc., Corning, NY, USA); cisplatin (Abmole Bioscience Inc., Houston, TX, USA); IL-6, IL-10, and TNF-α detection reagents (Servicebio Biotechnology Co., Ltd., Wuhan, China); methanol (purity ≥ 99.0%) and acetonitrile (purity 99.9%, Thermo Fisher Scientific, Waltham, MA, USA); chloroform (Wokai Biotechnology Co., Ltd., Beijing, China); 2-chloro-L-phenylalanine (Aladdin, Shanghai, China); RIPA lysis buffer, along with a phosphatase inhibitor and PMSF protease inhibitor (Solarbio, Beijing, China).

The following antibodies were utilized in this study: anti-MAPK1 (from CST, Inc., Danvers, MA, USA); anti-GRB2 and anti-PIK3R2 (sourced from Proteintech, Wuhan, China).

### 4.2. Research Animals

Sixty SPF female nude mice of the BALB/c strain, at an age of 4 to 6 weeks and each weighing roughly 20 g, were obtained from SJA Laboratory Animal Co., Ltd., Hunan, China (License No.: SCXK (Xiang) 2021-0002). The animals were housed under controlled conditions at the Animal Experiment Center of Xinjiang Medical University; this included maintaining a temperature of 24 ± 2 °C and a humidity level of 40–60%, and an SPF environment. All animals underwent a one-week acclimatization period prior to the experiments. The Animal Ethics Committee of Xinjiang Medical University approved all experimental procedures (Approval No. IACUC-JT-20250114-11, dated 14 January 2025).

### 4.3. Preparation of HeLa Cell Suspension

Human cervical cancer HeLa cells sourced from Procell Life Science & Technology Co., Ltd. in Wuhan, China were propagated in high-glucose DMEM complete medium, enriched with 10% fetal bovine serum, along with 100 U·mL^−1^ of penicillin and 100 µg·mL^−1^ of streptomycin. The cells were kept in a humidified incubator set at 37 °C with an atmosphere containing 5% CO_2_. After attaining 80–90% confluence, the cells were detached using 0.25% trypsin for 2 min, followed by the preparation of a cell suspension with PBS and Matrigel at a 1:1 ratio. The cell density was ultimately set to 6 × 10^6^ cells per milliliter.

### 4.4. Development of a Cervical Cancer Xenograft Model Using Athymic Mice

Sixty athymic BALB/c mice was utilized in the study, with ten allocated randomly to the untreated control group. The other fifty mice were utilized to develop the cervical cancer xenograft model. Each mouse had the right axilla injection site disinfected using a 75% alcohol swab before receiving a subcutaneous inoculation of 0.1 mL of the HeLa cell suspension. Subsequently, the mice were observed daily for overall well-being, encompassing mental status, food consumption, mobility, and tumor progression.

### 4.5. Grouping and Administration

Five days after successful tumor engraftment, indicated by a palpable subcutaneous nodule, the tumor-bearing mice were randomly assigned to five groups (n = 10 per group): the model group, the positive control group (cisplatin group), and low-, medium-, and high-dose gossypol groups. Gossypol was administered intragastrically to the low-, medium-, and high-dose gossypol groups at dosages of 10, 15, and 20 mg·kg^−1^, respectively, with a daily volume of 0.2 mL per mouse. Both the control group and the model group received an identical daily amount of normal saline. In contrast, the positive control group received intraperitoneal injections of cisplatin (at a dose of 2 mg·kg^−1^, diluted in 1% DMSO) administered at a dose of 0.2 mL for each mouse every other day. This treatment protocol was carried out for a total of 14 consecutive days, after which all mice were euthanized on day 15 using cervical dislocation while under anesthesia.

### 4.6. Assessment of Mouse Body Weight, Tumor Volume and Weight

These animals were monitored daily for their general condition and mental status, while body weight and tumor dimensions were measured every two days interval. The length (a) and width (b) of the tumor were measured and recorded, with the relative volume determined using the formula V = ab^2^/2. Twenty-four hours following the last administration, the mice were sedated using pentobarbital sodium, and approximately 1 mL of blood was obtained via orbital sampling. The blood collections were allowed to stand at room temperature for one hour before being centrifuged at 3000 rpm for 20 min at 4 °C to obtain the serum. Subsequently, the heart, liver, spleen, kidneys, and tumor tissues were excised, rinsed with saline, dried, and weighed to determine the organ index and tumor inhibition rate.

### 4.7. Histopathological Examination via HE Staining

Organs and tumor tissues from three randomly selected mice per group were preserved in 4% paraformaldehyde, incorporated in paraffin, sliced into sections, deparaffinized, and then rehydrated, and stained with HE following established procedures. Subsequently, the specimens were dehydrated, mounted, and microscopically evaluated for pathological alterations in different tissues within each group.

### 4.8. ELISA for Serum IL-6, IL-10, and TNF-α Levels

The levels of IL-6, IL-10, and TNF-α in the collected serum samples were measured using commercial ELISA kits, following the manufacturer’s instructions.

### 4.9. Analysis via Western Blot

Tumor tissues from the mice were harvested and homogenized using RIPA lysis buffer encompassing protease and phosphatase inhibitors, utilizing grinding beads, and subsequently subjected to centrifugation; entrifugate was then collected. Protein concentration was evaluated with the BCA assay. The protein samples were diluted with loading buffer, denatured at 100 °C for 10 min, and stored at −20 °C until needed. Proteins were first separated using 10% SDS-PAGE electrophoresis and then moved to PVDF membranes. The membranes were treated with 5% skim milk to block non-specific binding for 2 h under standard temperature conditions, before being treated with primary antibodies and kept at 4 °C overnight. Following the TBST wash, the membranes underwent incubation at room temperature for 1 h with corresponding antibodies of the second type (Proteintech, Wuhan, China). After conducting another round of washes with TBST, the protein fractions were revealed by means of an ECL chemiluminescence kit. β-Actin was utilized as the internal control for loading normalization, and the bands’ grayscale values were quantified employing ImageJ software (version 1.54p, NIH, Bethesda, MD, USA).

### 4.10. Untargeted Metabolomics Analysis

Liver tissue samples were homogenized in a solvent mixture comprising 75% (9:1 methanol to chloroform) and 25% water, subsequently subjected to ultrasonication and then centrifuged. The liquid extract was gathered, purified, and evaporated. The resulting residue was subsequently dissolved in a 50% acetonitrile solution containing 4 ppm of 2-chloro-L-phenylalanine, which served as the internal standard. After filtration, the samples underwent LC-MS analysis.

Metabolomic profiling was conducted utilizing an ultra-high-performance liquid chromatography system, the Thermo Vanquish (Thermo Fisher Scientific, Waltham, MA, USA), which was utilized for the analysis in conjunction with a Thermo Orbitrap Exploris 120 mass spectrometer (Thermo Fisher Scientific, Waltham, MA, USA). Separation was performed using an ACQUITY UPLC^®^ HSS T3 column (2.1 × 100 mm, 1.8 µm; Waters, Milford, MA, USA) kept at a temperature of 40 °C. The volumetric flow rate was set at 0.3 mL·min^−1^, and a 5 µL injection volume was used. In positive ion configuration, the mobile phase comprised 0.1% formic acid in acetonitrile (B2) and 0.1% formic acid in water (A2). In the case of negative ion mode, acetonitrile (B3) and 5 mM ammonium formate in water (A3) were employed. The elution gradient for both modes was as follows: 0–1 min, 10% B; 1–5 min, 10% to 98% B; 5–6.5 min, 98% B; 6.5–6.6 min, 98% to 10% B; 6.6–8 min, 10% B. Mass spectrometry detection was performed using an electrospray ionization (ESI) source. The spray voltage for the electropositive ion mode was established at 3.50 kV, whereas the voltage for the electronegative ion mode was configured to −2.50 kV. The flow rates for the sheath and auxiliary gases were set at 40 arb and 10 arb, in that order, while the capillary’s temperature recorded was held constant at 325 °C. Full-scan MS data were collected within the m/z range of 100–1000 at a resolution of 60,000. Data-dependent MS/MS analysis utilized higher-energy collisional dissociation (HCD) with a normalized collision energy of 30%. The four strongest precursor ions were chosen for fragmentation, and mass spectral data were collected at a resolution of 15,000; dynamic exclusion was enabled to avoid unnecessary fragmentation.

The raw mass spectrometry data files were transformed into mzXML format using ProteoWizard MSConvert software (version 3.0.8789). Following this conversion, peak detection, filtering, and alignment were performed with the XCMS package (version 3.12.0) in R to produce a quantitative metabolite feature table, which was then normalized based on total peak area. The identification of metabolites involved matching acquired spectra against various online repositories, including Massbank, HMDB, mzCloud, LipidMaps, KEGG, and a proprietary standard library (Nomi Metabolomics), with a mass tolerance of less than 15 ppm.

Various multivariate statistical methods were employed, including principal component analysis (PCA), partial least squares discriminant analysis (PLS-DA), and orthogonal partial least squares discriminant analysis (OPLS-DA) were performed using the Ropls package within the R programming environment. The effectiveness of the models was assessed by analyzing R2X, R2Y, and Q2 values, where values approaching 1 indicate higher explanatory and predictive capabilities. Additionally, a permutation test was executed to assess the risk of overfitting. To identify significantly altered metabolites, a comprehensive approach combining the *p*-value from Student’s *t*-test, the VIP score from the OPLS-DA model, and the fold change was employed. Metabolites that satisfied the thresholds of VIP greater than 1 and a *p*-value less than 0.05 were classified as statistically notable differential metabolites.

### 4.11. Data Analysis

Data analysis and visual presentation were conducted using GraphPad Prism software, specifically version 10.1.2. Comparative analysis was conducted between the two groups through an independent samples from Student’s *t*-test. For multiple groups, ANOVA was employed to assess differences. A *p*-value of less than 0.05 (*p* < 0.05) was deemed statistically significant.

### 4.12. Network Toxicology Investigation and Molecular Docking

The canonical structure and SMILES notation of gossypol were gathered from the PubChem database (https://pubchem.ncbi.nlm.nih.gov/ (accessed on 24 June 2025)). Potential drug targets were predicted utilizing the PharmMapper (http://www.lilab-ecust.cn/pharmmapper/ (accessed on 24 June 2025)), SwissTargetPrediction (http://www.swisstargetprediction.ch/ (accessed on 24 June 2025)), and STITCH (http://stitch.embl.de/ (accessed on 24 June 2025)) databases. The results were integrated and deduplicated to create a comprehensive list of targets. Simultaneously, targets associated with hepatotoxicity were gathered by searching the GeneCards (https://www.genecards.org (accessed on 24 June 2025)) and OMIM (https://www.omim.org/ (accessed on 24 June 2025)) databases with the keywords “hepatic injury,” “hepatotoxicity,” and “hepatic dysfunction.” The retrieved targets were also pooled and deduplicated. The intersecting targets between gossypol and hepatotoxicity were identified using Venny 2.1.0.

The PPI network was established through the use of Cytoscape software (version 3.10.0), focusing on the overlapping targets. Hub targets were identified utilizing the CytoHubba plugin. Subsequently, the intersecting targets were placed under KEGG pathway and GO analyses using the Metascape platform (https://metascape.org (accessed on 25 June 2025)) for functional enrichment.

Molecular docking was carried out with the hub targets identified by CytoHubba. The three-dimensional (3D) structures of the target proteins were retrieved from the RCSB Protein Data Bank (https://www.rcsb.org (accessed on 28 June 2025)), while the 3D structure of gossypol was obtained from the PubChem database and transformed into PDB structure utilizing Open Babel software, version 3.1.1. Docking simulations were carried out using the CB-Dock2 online platform, accessible at https://cadd.labshare.cn/cb-dock2/php/index.php (accessed on 28 June 2025), and the optimal binding pose for each docking result was visualized and rendered with PyMOL software (version 3.0.4).

## 5. Conclusions

Gossypol significantly suppressed tumor growth in a mouse model of cervical cancer in vivo and exerted its anti-cancer effects by inhibiting the expression of PIK3R2, GRB2, and MAPK1 proteins. Through integrated analysis using untargeted metabolomics to profile relevant metabolites and network toxicology to identify core targets, this study has preliminarily elucidated the mechanisms underlying gossypol-induced hepatotoxicity during its therapeutic application for cervical cancer. These findings hold significant implications for the progression of therapies targeting cervical cancer. Nonetheless, the exact mechanisms of action responsible for gossypol-induced liver injury require further in-depth experimental validation in subsequent studies. Furthermore, HIF-1α serves as a core regulator of cervical cancer advancement in hypoxic niches and is known to interact with the PI3K/AKT pathway. A critical yet unexplored question is whether gossypol influences the HIF-1α signaling axis to mediate its effects on tumor metabolic reprogramming, angiogenesis, and adaptation to hypoxia, representing a promising avenue for further investigation.

## Figures and Tables

**Table 1:** Tumor growth inhibition rates in mice from different treatment groups ($\bar{x}$ ± s, *n* = 6).

Group	Dose	Tumor Weight (g)	Tumor Inhibition Rate (%)
Model (MOD)	-	0.732 ± 0.060	-
Cisplatin (DDP)	2 mg·kg^−1^	0.310 ± 0.072 ****	57.63%
Low-dose gossypol (GPL)	10 mg·kg^−1^	0.650 ± 0.052	11.16%
Medium-dose gossypol (GPM)	15 mg·kg^−1^	0.448 ± 0.050 ****	38.72%
High-dose gossypol (GPH)	20 mg·kg^−1^	0.393 ± 0.115 ****	46.24%

**** *p* < 0.0001, vs. MOD.

**Table 2:** Significantly differential metabolites in mouse liver tissue.

No.	Name	Formula	*m*/*z*	tR (s)	VIP	*p* Value
1	(3E)-3-[1-amino-3-methyl-5-[(E)-2-methyltetradec-4-en-6,8-diynyl]pyrrolidin-2-ylidene]-1,5-dimethylpyrrolidine-2,4-dione	C_26_H_37_N_3_O_2_	446.28	336.1	1.240	0.010228
2	4,4-Diapolycopenedial	C_30_H_36_O_2_	473.2677	362	1.015	0.006229
3	6-Phosphogluconic Acid	C_6_H_13_O_10_P	275.017	42.2	1.066	0.000156
4	7a-Hydroxydehydroepiandrosterone	C_19_H_28_O_3_	349.2016	314.5	1.143	0.000082
5	11-Methyl-7-oxatetracyclo[6.3.1.01,6.04,11]dodecane	C_12_H_18_O	179.1434	365.2	1.041	0.007600
6	Acetoacetyl-CoA	C_25_H_40_N_7_O_18_P_3_S	835.1207	229.2	1.280	0.000328
7	Deoxymyxol	C_40_H_56_O_2_	552.4108	414.1	1.301	0.009319
8	Isoxanthohumol	C_21_H_22_O_5_	353.1415	445.2	1.099	0.000504
9	Timonacic	C_4_H_7_NO_2_S	134.0274	54.3	1.024	0.000240

**Table 3:** Molecular docking binding energies between gossypol and its potential targets.

No.	Protein Name	PDB ID	Binding Energy (kJ·mol^−1^)
1	mTOR	4DRI	−9.8
2	ALB	6YG9	−8.0
3	TNF	5UUI	−6.8
4	ESR1	7BAA	−6.4
5	HSP90AB1	6N8Y	−6.5
6	CTNNB1	3FQN	−8.9
7	CASP3	2DKO	−7.4
8	BCL2L1	7JGW	−8.2
9	BCL2	8HTS	−7.1
10	HSP90AA1	5J80	−7.9

## Data Availability

The original contributions presented in this study are included in the article. Further inquiries can be directed to the corresponding author.

## References

[1] World Health Organization https://www.who.int/news-room/fact-sheets/detail/cervical-cancer.

[2] Xu M., Cao C., Wu P., Huang X., Ma D. (2025). Advances in cervical cancer: Current insights and future directions. Cancer Commun..

[3] Zhou J., Guo Z., Peng X., Wu B., Meng Q., Lu X., Feng L., Guo T. (2025). Chrysotoxine regulates ferroptosis and the PI3K/AKT/mTOR pathway to prevent cervical cancer. J. Ethnopharmacol..

[4] Siegel R.L., Giaquinto A.N., Jemal A. (2024). Cancer statistics, 2024. CA Cancer J. Clin.

[5] Yao S., Chen S., Wang A., Liang Z., Liu X., Gao Y., Cai H. (2025). BAG2 Inhibits Cervical Cancer Progression by Modulating Type I Interferon Signaling through Stabilizing STING. Adv. Sci..

[6] Sun X., Ying J., Ma X., Zhong Y., Huo R., Meng Q. (2025). Effects of Gossypol Exposure on Ovarian Reserve Function: Comprehensive Risk Assessment Based on TRAEC Strategy. Toxics.

[7] Radloff R.J., Deck L.M., Royer R.E., Vander Jagt D.L. (1986). Antiviral activities of gossypol and its derivatives against herpes simplex virus type II. Pharmacol. Res. Commun..

[8] Keshmiri-Neghab H., Goliaei B. (2014). Therapeutic potential of gossypol: An overview. Pharm. Biol..

[9] Chirawurah J.D., Ansah F., Blankson S., Adikah B., Yeboah S.N., Amenga-Etego L., Awandare G.A., Aniweh Y. (2025). Gossypol is a natural product with good antimalarial activity against Plasmodium falciparum clinical isolates. Sci. Rep..

[10] Hilliard A.L., Russell T.D., Mendonca P., Soliman K.F.A. (2025). Targeting the Tumor Immune Microenvironment in Triple-Negative Breast Cancer: The Promise of Polyphenols. Cancers.

[11] Lopez-Charcas O., Benouna O., Lemoine R., Rosendo-Pineda M.J., Anguheven-Ledezma T.G., Sandoval-Vazquez L., Gallegos-Gomez M.L., Robles-Martinez L., Herrera-Carrillo Z., Ramírez-Aragón M. (2024). Blockade of Ca(V)3 calcium channels and induction of G(0)/G(1) cell cycle arrest in colon cancer cells by gossypol. Br. J. Pharmacol..

[12] Paunovic D., Rajkovic J., Novakovic R., Grujic-Milanovic J., Mekky R.H., Popa D., Calina D., Sharifi-Rad J. (2023). The potential roles of gossypol as anticancer agent: Advances and future directions. Chin. Med..

[13] Stepanov A.V., Yarovenko V.N., Nasyrova D.I., Dezhenkova L.G., Akchurin I.O., Krayushkin M.M., Ilyushenkova V.V., Shchekotikhin A.E., Tretyakov E.V. (2024). A Spin-Labeled Derivative of Gossypol. Molecules.

[14] Hu M., Zheng K., Zhang L., Kan Y., Zhao J., Chen D. (2025). Therapeutic Strategies Targeting Aerobic Glycolysis in Cancer and Dynamic Monitoring of Associated Metabolites. Cells.

[15] Hsieh Y.S., Chu S.C., Huang S.C., Kao S.H., Lin M.S., Chen P.N. (2021). Gossypol Reduces Metastasis and Epithelial-Mesenchymal Transition by Targeting Protease in Human Cervical Cancer. Am. J. Chin. Med..

[16] Li Y., Qu J., Liu L., Sun Y., Zhang J., Han S., Zhang Y. (2022). Apogossypolone Inhibits Cell Proliferation and Epithelial-Mesenchymal Transition in Cervical Cancer via Activating DKK3. Front. Oncol..

[17] Ma C., Lu X., Ni C., Gao Y., Yang F., Chen S., Du Y., Zhao F., Cao Y., Huang H. (2025). SLC25A10 promotes cisplatin resistance by inhibiting ferroptosis in cervical cancer. Cell Death Discov..

[18] Ye M., Liu T., Miao L., Ji H., Xu Z., Wang H., Zhang J., Zhu X. (2024). Cisplatin-encapsulated TRAIL-engineered exosomes from human chorion-derived MSCs for targeted cervical cancer therapy. Stem Cell Res. Ther..

[19] LI J., Asat R., Li W., Parhat P., Ma Y., Ma Y., Li M. (2025). Molecular Target Identification of Gossypol Against Cervical Cancer Based on Target Fishing Technology. Pharmaceutics.

[20] Gu Y., Yang M., Wang W., Li L., Ma Y., Liu W., Zhao Q. (2025). YX-112, a novel celastrol-derived PROTAC, inhibits the development of triple-negative breast cancer by targeting the degradation of multiple proteins. Front. Pharmacol..

[21] Scribano D., Tito C., Tagueha A.D., Pasqua M., De Angelis L., Fazi F., Limongi D., De Angelis M., Nencioni L., Palamara A.T. (2025). Goblet cell breakdown: Transcriptomics reveals Acinetobacter baumannii early and robust inflammatory response in differentiated human bronchial epithelial cells. J. Biomed. Sci..

[22] Akutagawa K., Miki S., Yamada E., Sakamoto N., Miyazaki T., Sugii N., Zaboronok A., Matsuda M., Ishikawa E. (2025). PIK3R2 immunostaining status predicts prognosis in patients with newly diagnosed glioblastoma treated with an autologous tumor vaccine. J. Neurooncol.

[23] De Geus V., Ewing-Graham P.C., De Koning W., De Koning M.N.C., Van Den Bosch T.P.P., Nigg A.L., Van Eijck C.H.J., Jozwiak M., Van Beekhuizen H.J., Mustafa D.A.M. (2021). Identifying Molecular Changes in Early Cervical Cancer Samples of Patients That Developed Metastasis. Front. Oncol..

[24] Tran D.N., Hwang Y.J., Kim K.C., Li R., Marquardt R.M., Chen C., Young S.L., Lessey B.A., Kim T.H., Cheon Y.P. (2025). GRB2 regulation of essential signaling pathways in the endometrium is critical for implantation and decidualization. Nat. Commun..

[25] Malagrinò F., Puglisi E., Pagano L., Travaglini-Allocatelli C., Toto A. (2024). GRB2: A dynamic adaptor protein orchestrating cellular signaling in health and disease. Biochem. Biophys. Rep..

[26] Yao L., Wang W., Zhang B. (2025). Exploring chamazulene as a novel therapeutic agent for breast cancer in silico and in vitro: Apoptosis induction, cell cycle regulation, and antimetastatic effects. Front. Pharmacol..

[27] Lin C.C., Kuo C.L., Huang Y.P., Chen C.Y., Hsu M.J., Chu Y.L., Chueh F.S., Chung J.G. (2018). Demethoxycurcumin Suppresses Migration and Invasion of Human Cervical Cancer HeLa Cells via Inhibition of NF-κB Pathways. Anticancer. Res..

[28] Lu M., Gao Q., Wang Y., Ren J., Zhang T. (2022). LINC00511 promotes cervical cancer progression by regulating the miR-497-5p/MAPK1 axis. Apoptosis.

[29] Zhang Y., Huang F., Zhai J., Sun J., Li B., Zhang S. (2025). Mechanism of Huaiqihuang (HQH) against cyclophosphamide (CYP)-induced hippocampal neurotoxicity based on network pharmacology, molecular docking and experimental verification. Front. Cell Dev. Biol..

[30] Yuan C., Wu J., Xiang Y., Ni L. (2025). Deciphering cellular heterogeneity and pathway dynamics in urinary samples: A UMAP-Based approach to understanding acute kidney injury. Front. Pharmacol..

[31] Jiang H., Liang M., Jiang Y., Zhang T., Mo K., Su S., Wang A., Zhu Y., Huang G., Zhou R. (2019). The lncRNA TDRG1 promotes cell proliferation, migration and invasion by targeting miR-326 to regulate MAPK1 expression in cervical cancer. Cancer Cell Int..

[32] Li X.W., Tuergan M., Abulizi G. (2015). Expression of MAPK1 in cervical cancer and effect of MAPK1 gene silencing on epithelial-mesenchymal transition, invasion and metastasis. Asian Pac. J. Trop. Med..

[33] Liu X., Yin Z., Wang Y., Cao S., Yao W., Liu J., Lu X., Wang F., Zhang G., Xiao Y. (2022). Rice cellulose synthase-like protein OsCSLD4 coordinates the trade-off between plant growth and defense. Front. Plant Sci..

[34] Li R., Wang W., Yang Y., Gu C. (2020). Exploring the role of glucose-6-phosphate dehydrogenase in cancer (Review). Oncol. Rep..

[35] Liu R., Li X., Xu J., Yan L., Hu K., Shi M., Zhang Y., Zhao Y., Fan Y., Wang G. (2025). The contrasting regulatory effects of valproic acid on ferroptosis and disulfidptosis in hepatocellular carcinoma. Theranostics.

[36] Mcgivney G.R., Brockman Q.R., Borcherding N., Scherer A., Rauckhorst A.J., Gutierrez W.R., Solst S.R., Heer C.D., Warrier A., Floyd W. (2025). Somatic CRISPR tumorigenesis and multiomic analysis reveal a pentose phosphate pathway disruption vulnerability in MPNSTs. Sci. Adv..

[37] Fang Z., Jiang C., Feng Y., Chen R., Lin X., Zhang Z., Han L., Chen X., Li H., Guo Y. (2016). Effects of G6PD activity inhibition on the viability, ROS generation and mechanical properties of cervical cancer cells. Biochim. Biophys. Acta.

[38] Beiv Y., Wang S., Wang R., Ahmad O., Jia M., Yao P., Ji J., Shen P. (2025). CDK5-triggered G6PD phosphorylation at threonine 91 facilitating redox homeostasis reveals a vulnerability in breast cancer. Acta Pharm. Sin. B.

[39] Rebelo A., Kleeff J., Sunami Y. (2023). Cholesterol Metabolism in Pancreatic Cancer. Cancers.

[40] Jezewski A.J., Esan T.E., Propp J., Fuller A.J., Daraji D.G., Lail C., Staker B.L., Woodward E.L., Liu L. (2024). A single Leishmania adenylate-forming enzyme of the ANL superfamily generates both acetyl- and acetoacetyl-CoA. J. Biol. Chem..

[41] Gao Y., Sheng X., Tan D., Kim S., Choi S., Paudel S., Lee T., Yan C., Tan M., Kim K.M. (2023). Identification of Histone Lysine Acetoacetylation as a Dynamic Post-Translational Modification Regulated by HBO1. Adv. Sci..

[42] Bergstrom J.D. (2023). The lipogenic enzyme acetoacetyl-CoA synthetase and ketone body utilization for denovo lipid synthesis, a review. J. Lipid Res..

[43] Aoyama T., Paik Y.H., Seki E. (2010). Toll-like receptor signaling and liver fibrosis. Gastroenterol. Res. Pract..

[44] Huang L., Gan L., Pan J., Zhong L., Wang Q., Luo S., Tian J., Liang H. (2022). Transcriptomics combined with metabolomics analysis of the mechanism of agmatine in the treatment of septic liver injury. Ann. Transl. Med..

[45] Li J.X., Han Z.X., Cheng X., Zhang F.L., Zhang J.Y., Su Z.J., Li B.P., Jiang Z.R., Li R.Z., Xie Y. (2023). Combinational study with network pharmacology, molecular docking and preliminary experiments on exploring common mechanisms underlying the effects of weijing decoction on various pulmonary diseases. Heliyon.

[46] Liu Z., Zhang H., Yao J. (2024). Metabolomic Profiling and Network Toxicology: Mechanistic Insights into Effect of Gossypol Acetate Isomers in Uterine Fibroids and Liver Injury. Pharmaceuticals.

[47] Stuhldreier F., Schmitt L., Lenz T., Hinxlage I., Zimmermann M., Wollnitzke P., Schliehe-Diecks J., Liu Y., Jäger P., Geyh S. (2022). The mycotoxin viriditoxin induces leukemia- and lymphoma-specific apoptosis by targeting mitochondrial metabolism. Cell Death Dis..

[48] Corcoran R.B., Do K.T., Kim J.E., Cleary J.M., Parikh A.R., Yeku O.O., Xiong N., Weekes C.D., Veneris J., Ahronian L.G. (2024). Phase I/II Study of Combined BCL-xL and MEK Inhibition with Navitoclax and Trametinib in KRAS or NRAS Mutant Advanced Solid Tumors. Clin. Cancer Res..

[49] Stuard Sambhariya W., Trautmann I.J., Robertson D.M. (2023). Insulin-like growth factor binding protein-3 mediates hyperosmolar stress-induced mitophagy through the mechanistic target of rapamycin. Biol. Chem..

[50] Zhou B.G., Zhao H.M., Lu X.Y., Zhou W., Liu F.C., Liu X.K., Liu D.Y. (2018). Effect of Puerarin Regulated mTOR Signaling Pathway in Experimental Liver Injury. Front. Pharmacol..

[51] Platt E., Klootwijk E., Salama A., Davidson B., Robertson F. (2022). Literature review of the mechanisms of acute kidney injury secondary to acute liver injury. World J. Nephrol..

[52] Mesarwi O.A., Shin M.K., Bevans-Fonti S., Schlesinger C., Shaw J., Polotsky V.Y. (2016). Hepatocyte Hypoxia Inducible Factor-1 Mediates the Development of Liver Fibrosis in a Mouse Model of Nonalcoholic Fatty Liver Disease. PLoS ONE.

[53] Cano-Gómez C.I., Alonso-Castro A.J., Carranza-Alvarez C., Wong-Paz J.E. (2024). Advancements in Litchi chinensis Peel Processing: A Scientific Review of Drying, Extraction, and Isolation of Its Bioactive Compounds. Foods.

[54] Lu L., Ma Y., Tao Q., Xie J., Liu X., Wu Y., Zhang Y., Xie X., Liu M., Jin Y. (2025). Hypoxia-inducible factor-1 alpha (HIF-1α) inhibitor AMSP-30 m attenuates CCl(4)-induced liver fibrosis in mice by inhibiting the sonic hedgehog pathway. Chem. Biol. Interact..

[55] Zhou J.C., Wang J.L., Ren H.Z., Shi X.L. (2022). Autophagy plays a double-edged sword role in liver diseases. J. Physiol. Biochem..

[56] Zhang Z., Wang J., Li H., Niu Q., Tao Y., Zhao X., Zeng Z., Dong H. (2025). The role of the interleukin family in liver fibrosis. Front. Immunol..

[57] Tang T.L., Yang Y., Guo L., Xia S., Zhang B., Yan M. (2022). Sunitinib induced hepatotoxicity in L02 cells via ROS-MAPKs signaling pathway. Front. Pharmacol..

[58] Dituri F., Gigante G., Scialpi R., Mancarella S., Fabregat I., Giannelli G. (2022). Proteoglycans in Cancer: Friends or Enemies? A Special Focus on Hepatocellular Carcinoma. Cancers.

[59] Yazici S.E., Gedik M.E., Leblebici C.B., Kosemehmetoglu K., Gunaydin G., Dogrul A.B. (2023). Can endocan serve as a molecular “hepatostat” in liver regeneration?. Mol. Med..

[60] Bai JChen Y., Ning Z., Liu S., Xu C., Yan J.K. (2020). Proteoglycan isolated from Corbicula fluminea exerts hepato-protective effects against alcohol-induced liver injury in mice. Int. J. Biol. Macromol..

